# Culturing oil sands microbes as mixed species communities enhances *ex situ* model naphthenic acid degradation

**DOI:** 10.3389/fmicb.2015.00936

**Published:** 2015-09-04

**Authors:** Marc A. Demeter, Joseph A. Lemire, Gordon Yue, Howard Ceri, Raymond J. Turner

**Affiliations:** Biofilm Research Group, Department of Biological Sciences, University of Calgary, CalgaryAB, Canada

**Keywords:** naphthenic acids, oil sands, bioremediation, biofilms, bioreactor

## Abstract

Oil sands surface mining for bitumen results in the formation of oil sands process water (OSPW), containing acutely toxic naphthenic acids (NAs). Potential exists for OSPW toxicity to be mitigated by aerobic degradation of the NAs by microorganisms indigenous to the oil sands tailings ponds, the success of which is dependent on the methods used to exploit the metabolisms of the environmental microbial community. Having hypothesized that the xenobiotic tolerant biofilm mode-of-life may represent a feasible way to harness environmental microbes for *ex situ* treatment of OSPW NAs, we aerobically grew OSPW microbes as single and mixed species biofilm and planktonic cultures under various conditions for the purpose of assaying their ability to tolerate and degrade NAs. The NAs evaluated were a diverse mixture of eight commercially available model compounds. Confocal microscopy confirmed the ability of mixed and single species OSPW cultures to grow as biofilms in the presence of the NAs evaluated. qPCR enumeration demonstrated that the addition of supplemental nutrients at concentrations of 1 g L^-1^ resulted in a more numerous population than 0.001 g L^-1^ supplementation by approximately 1 order of magnitude. GC-FID analysis revealed that mixed species cultures (regardless of the mode of growth) are the most effective at degrading the NAs tested. All constituent NAs evaluated were degraded below detectable limits with the exception of 1-adamantane carboxylic acid (ACA); subsequent experimentation with ACA as the sole NA also failed to exhibit degradation of this compound. Single species cultures degraded select few NA compounds. The degradation trends highlighted many structure-persistence relationships among the eight NAs tested, demonstrating the effect of side chain configuration and alkyl branching on compound recalcitrance. Of all the isolates, the *Rhodococcus* spp. degraded the greatest number of NA compounds, although still less than the mixed species cultures. Overall, these observations lend support to the notion that harnessing a community of microorganisms as opposed to targeted isolates can enhance NA degradation *ex situ*. Moreover, the variable success caused by NA structure related persistence emphasized the difficulties associated with employing bioremediation to treat complex, undefined mixtures of toxicants such as OSPW NAs.

## Introduction

A rapidly growing source of unconventional petroleum, the oil sands of the Athabasca basin (located primarily in Alberta, Canada) contain an estimated 1.7 trillion barrels of bitumen (Energy Resources Conservation Board [ERCB], 2010). Recovery of bituminous ore from oil sands is depth dependent, with surface mining operations of shallow oil sands deposits utilizing a water intensive, caustic hot water process – the Clark Extraction Process. This process results in the formation of large volumes of both semi-solid tailings waste (mature fine tailings), and an aqueous tailings waste known as oil sands process water (OSPW). OSPW contains organic and inorganic contaminants of environmental concern, including naphthenic acids (NAs), polycyclic aromatic hydrocarbons (PAHs), naphtha diluents and heavy metals (Nix and Martin, 1992; Kannel and Gan, 2012; Abolfazlzadehdoshanbehbazari et al., 2013). As reviewed by Allen (2008a), OSPW is capable of exerting both acute and chronic toxicity to a variety of aquatic organisms and mammals. The toxicological potential, along with the absence of a viable treatment option has led the Alberta government to legislate a zero discharge policy for OSPW, resulting in a large-scale buildup of tailings. NAs have been identified as a primary source of acute toxicity (Lo et al., 2006; Goff et al., 2013), and as a result are often the focus of potential OSPW remediation strategies.

The biodegradability of NAs by OSPW microorganisms has been explored and reviewed extensively (Clemente and Fedorak, 2005; Quagraine et al., 2005; Allen, 2008b; Kannel and Gan, 2012), but no one particular method to harness the naturally occurring microbes for use in *ex situ* NA treatment has been established and put into practice. One potential strategy that has been proposed and evaluated in a limited number of studies is the use of microbial biofilms in *ex situ* bioreactors targeting NA bioremediation (Headley et al., 2010; Huang et al., 2012; Gunawan et al., 2014; McKenzie et al., 2014).

Defined as a sessile aggregation of bacterial cells encapsulated in an exopolymeric matrix (Hall-Stoodley et al., 2004), biofilms are an attractive method for bioremediation purposes (Stach and Burns, 2002; Edwards and Kjellerup, 2013). As opposed to the planktonic (free-swimming) mode-of-life, biofilms exhibit enhanced mechanical stability, which may serve to retain biomass in a bioreactor (Lendenmann and Spain, 1998; Nicolella et al., 2000). Moreover, biofilms are more resistant to a variety of toxic compounds via biofilm specific mechanisms including: mass transfer resistant concentration boundary layers (Stewart, 2012), limited toxicant penetration due to diffusion-reactions with biofilm matrix components (Stewart et al., 2001; van Hullebusch et al., 2003; Chambless et al., 2006), and active or stimulatory toxicant responses such as quorum sensing (García-Contreras et al., 2014), or the stringent response (Nguyen et al., 2011). Furthermore, superior rates of horizontal gene transfer in biofilms (Hausner and Wuertz, 1999) have the potential to enhance xenobiotic degradation gene propagation (Dejonghe et al., 2000). Lastly, the close association of cells within a biofilm has been demonstrated to promote intercellular social interactions that accelerate the use of xenobiotic substrates (Chakraborty et al., 2012).

In a prior study, our group successfully cultured community biofilms directly from oil sands tailings using the Calgary Biofilm Device (CBD) (Golby et al., 2012). This work was notable for demonstrating that this particular method allowed for the simultaneous cultivation of ∼80% of the complex microbial community indigenous to oil sands tailings, vastly outperforming traditional agar plate methods for *in vitro* harnessing of microbes from their environment – a phenomenon often described as the great plate count anomaly. Golby et al. (2012) also demonstrated that unique, targeted sub-populations of the whole oil sands tailings community could be achieved by simple manipulation of abiotic factors such as oxygen tension and nutrient amendment. Subsequent study of the representative environmental mixed species biofilms grown under these controlled, reproducible laboratory conditions in the CBD demonstrated oil sands tailings-derived biofilms could degrade two simple NAs [cyclohexane carboxylic acid (CHCA), and cyclohexane acetic acid (CHAA); Demeter et al., 2014]. Combined, this led to our working hypothesis that we can harness an OSPW microbial community with the metabolic potential to aerobically remove NA compounds from OSPW, and that for the reasons described above, culturing as a multispecies biofilm will prove the most efficacious method to harness these microbes with the goal of degrading NAs *ex situ*. The study herein evaluates NAs with greater variability in the degrees of compound recalcitrance and toxicity by including up to eight NAs with different extents of cyclization, alkyl side chain branching, and hydrophobicity as a controlled proxy for OSPW acid extractable components. In this study, an OSPW sample was used to grow single and mixed species planktonic and biofilm cultures using microtiter plates and the CBD respectively. Cultures were evaluated (via gas chromatography) for their ability to degrade the mixture of eight model NAs. Cultivation conditions were varied with respect to additional carbon sources in an attempt to elucidate appropriate supplement sources and concentrations to optimize metabolic potential in an up-scaled bioreactor.

## Materials and Methods

### Bacterial Inoculant

All bacterial inoculants were sourced from a sole Alberta OSPW sample following established methods (Demeter et al., 2014). The multispecies biofilm and planktonic cultivations received the diverse OSPW community via direct inoculation with raw OSPW. In order to obtain individual species for OSPW relevant evaluations, 100 μL of OSPW was spread onto minimal media agar plates [modified Bushnell-Haas (BH) media enriched with either glucose or yeast extract at 1 g L^-1^] along with two model NAs (CHCA and CHAA at 100 mg L^-1^ total NA concentration) and incubated at 25°C for up to 21 days. This procedure yielded six single colony isolates identified by unidirectional sequencing and BLAST (http://blast.ncbi.nlm.nih.gov/Blast.cgi) alignment of the 16S rRNA gene (**Table 1**). Single species biofilm and planktonic cultivations were subsequently inoculated with one of these six distinct isolates.

**Table 1:** Isolates obtained from the OSPW community.

Isolation medium	Taxonomy	Abbreviations	GenBank accession number
Bushnell-Haas	*Cyanobacteria*	G1	KT156639
Glucose	*Ancylobacter* sp.	G2	KT156640
(BH-G)	*Rhodococcus* sp.	G3	KT156641
Bushnell-Haas	*Xanthobacter* sp.	Y1	KT156642
Yeast extract	*Pseudomonas* sp.	Y2	KT156643
(BH-Y)	*Rhodococcus* sp.	Y3	KT156644

*Abbreviations are provided to identify the isolation carbon source in-text.*

### Naphthenic Acids Tested

As a better-defined and more consistent proxy to the NA fraction of OSPW, a synthetic mixture of eight commercially available NAs was prepared as sodium salt naphthenates and incorporated into the BH media at a working NA concentration of 200 mg L^-1^ (unless stated otherwise). This concentration is specifically chosen to accommodate increasing concentrations of NAs in oil sands tailings (currently reported at 40–120 mg L^-1^) as water is recycled in the extraction process (Quagraine et al., 2005). Following previously used nomenclature (Demeter et al., 2014), this mixture is referred to as 8χNA, and contains equimolar (1.7 mM) amounts of the NAs shown in **Table 2** (all purchased from Sigma-Aldrich). In a smaller, targeted assay, ACA, at a working concentration of 100 mg L^-1^ was used as the sole NA evaluated. Sodium salt naphthenates are soluble in water (Clemente and Fedorak, 2005).

**Table 2:** Constituent model NAs contained within the 8χNA mixture at equimolar (1.7 mM) concentrations.

Naphthenic acid	Abbreviations	Structure
Cyclohexane carboxylic acid	CHCA	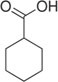
Cyclohexane acetic acid	CHAA	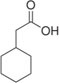
Hexanoic acid	HA	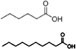
Decanoic acid	DA	
3-Methyl-1-cyclohexane carboxylic acid (*cis* and *trans*)	mCHCA	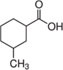
1-Adamantane carboxylic acid	ACA	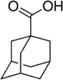
Cyclohexane butyric acid	CHBA	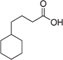
		
Cyclopentane carboxylic acid	CPCA	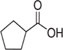

### Bacterial Cultivation and Growth Conditions

The bacterial growth media and cultivation techniques used are aerobic, and mimic that of previous work from our group (Golby et al., 2012; Demeter et al., 2014). Viability control multispecies cultures were grown in trypticase soy broth (TSB; Difco), while all other cultures were grown in a modified BH minimal salts medium (Wyndham and Costerton, 1981; Demeter et al., 2014), containing the 8χNA mixture, and supplemented with either 1 or 0.001 g L^-1^ glucose, yeast extract, peptone, or molasses as a stimulatory carbon source complementary to the 8χNA. Un inoculated media served as sterile controls for both biofilm and planktonic growth.

All biofilms used in this study were grown using the CBD; a specialized reaction vessel in which equivalent biofilms form on the pegs immersed in the medium contents of a standard 96 – well microtiter plate (Ceri et al., 1999; Harrison et al., 2010). Biofilms were grown in the CBD in the manner described earlier (Demeter et al., 2014). Inoculation of multispecies biofilms occurred by mixing OSPW with twice concentrated BH media in a 1:1 ratio for a total volume of 150 μL per well of the microtiter plate. Single species biofilms were inoculated with BH medium (150 μL) containing a 1 in 30 dilution of a 1.0 McFarland standard (i.e., ∼10^7^ cfu mL^-1^) of the appropriate isolate. Two days after the initial inoculation, biofilms were replenished with 150 μL of fresh medium, clearing the CBD system of inoculum cells that failed to adhere to the device peg, along with naturally occurring NAs from the OSPW inoculum that would interfere with the NA degradation assays.

Planktonic cultures were grown in standard 96 – well microtiter plates. Multispecies planktonic cultures were inoculated with 20 μL (per well) of the respective biofilm growth condition spent media (obtained upon biofilm media replenishment) along with 130 μL of the appropriate BH media. Rationalization for preparing multispecies planktonic cultures in this manner is provided in Demeter et al. (2014). Single species planktonic cultures were inoculated as described above for single species biofilm cultures.

Upon inoculation, both the CBD device, and planktonic 96 – well plates were incubated aerobically at 25°C, on a shaker set at 125 rpm for a total of 14 days.

### Bacterial Growth Evaluation

Biofilm growth was confirmed by use of confocal laser scanning microscopy (CLSM). Prior to visualization using a Leica DM IRE2 microscope equipped with a 64x water immersion objective and a Texas Red filter, the biofilms were stained with Syto 61 (Invitrogen, USA; Harrison et al., 2006; Golby et al., 2012; Demeter et al., 2014). Images were processed using Imaris x64 (Bitplane, USA). Planktonic growth was confirmed by measuring optical density at 450, 550, and 650 nm.

In order to semi-quantify both biofilm and planktonic cultures, genomic DNA was extracted using bead-beater methods (Golby et al., 2012; Schwering et al., 2013) and analyzed for prokaryotic 16S rRNA genes via quantitative PCR (qPCR). The qPCR method utilized a Rotor – Gene qPCR machine (Qiagen, Canada), universal 16S rRNA gene primers 926f and 1392r, a SYBR Green (Qiagen) detection system and a standard curve generated using genomic DNA from *Pseudomonas fluorescens* Pf – 5 (ATCC 13525). Specific details of this method, including the PCR program and the formulae used to determine 16S rRNA gene copy numbers are available in our prior study (Demeter et al., 2014). 16S rRNA gene copy numbers are reported in units relevant to one CBD chamber, allowing for a direct comparison; biofilm quantification is reported as 16S rRNA gene copies per peg, whereas planktonic quantification is reported as 16S rRNA gene copies per well.

### NA Degradation Analysis

Upon completion of the 14 days incubation period, spent media from eight identical microtiter wells (1.2 mL) along with 100 μL of the internal standard 4-phenyl butyric acid (4-PBA) at 1.3 g L^-1^ (Sigma-Aldrich) was collected in sterile 1.5 mL microfuge tubes, acidified to pH = 2 with 5.2 M HCl, and transferred to two dram glass vials with Teflon-lined lids for extraction into two volumes of dichloromethane. The organic phase containing the NAs was collected using phase separating filter papers (GE Whatman), and condensed with a rotary evaporator, after which the NAs are derivatized into trimethylsilylates using 150 μL of *N,O*-bis(trimethylsilyl)trifluoroacetamide (Fluka; Quesnel et al., 2011). The abundance (relative to the internal standard) of each constituent NA within the 8χNA mixture was then determined for each sample by use of gas chromatography coupled to a flame ionization detector (GC-FID). The GC-FID (Agilent 7890 model) utilized an Agilent HP-5 30 m column, operating with an injection volume of 4 μL, an injector split ratio of 2:1, and the following oven temperature program: 2 min at 70°C, followed by an increase at a ramp rate of 5°C min^-1^ until reaching 230°C (held for 2 min). Within this system, individual NA retention times (min) were as follows, CHCA = 10.0; CHAA = 12.5; HA = 6.9; DA = 16.6; mCHCA = 10.8/11.5; ACA = 19.0; CHBA = 17.8; CPCA = 7.7; and 4-PBA = 18.0 min. Day 0 (initial 8χNA) levels were also determined by extracting NAs from fresh 8χNA media, and used to normalize the relative abundance of each constituent NA as a fraction of the starting amount. Sterile controls were analyzed for abiotic loss of NAs. It is important to note the methods used in this study can only detect loss or change of the parent structure. Any observed NA degradation was in fact a loss of the parent structure, and not necessarily mineralization of the NA in question.

### Data Evaluation

Data from duplicate or triplicate incubations was reported throughout this study as a mean with standard error. No claims of statistical significance were made as no statistical tests were employed.

## Results

### OSPW Microbial Growth in the Presence of 8χNA Mixture

**FIGURE 1:**
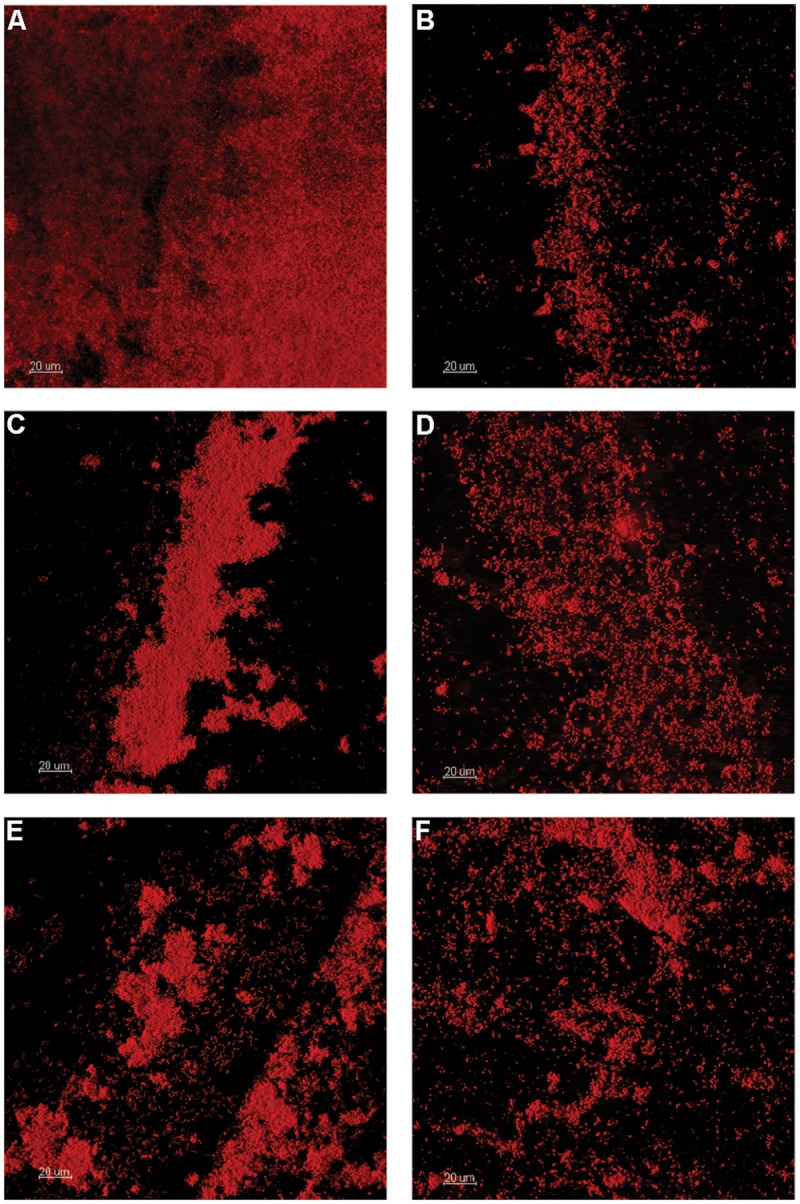
**Confocal laser scanning microscopy (CLSM) of OSPW multispecies biofilms grown on the polystyrene pegs of the CBD for 14 days at 25°C, and 125 rpm.** TSB rich medium was inoculated with OSPW to serve as a control for inoculum viability **(A)**. The remaining biofilms were grown in the presence of 8χNA, using BH minimal media supplemented with 0.001 g L^-1^ molasses **(B)**, 1 g L^-1^ yeast extract **(C)**, 0.001 g L^-1^ yeast extract **(D)**, 1 g L^-1^ peptone **(E)**, and 0.001 g L^-1^ peptone **(F)**.

**FIGURE 2:**
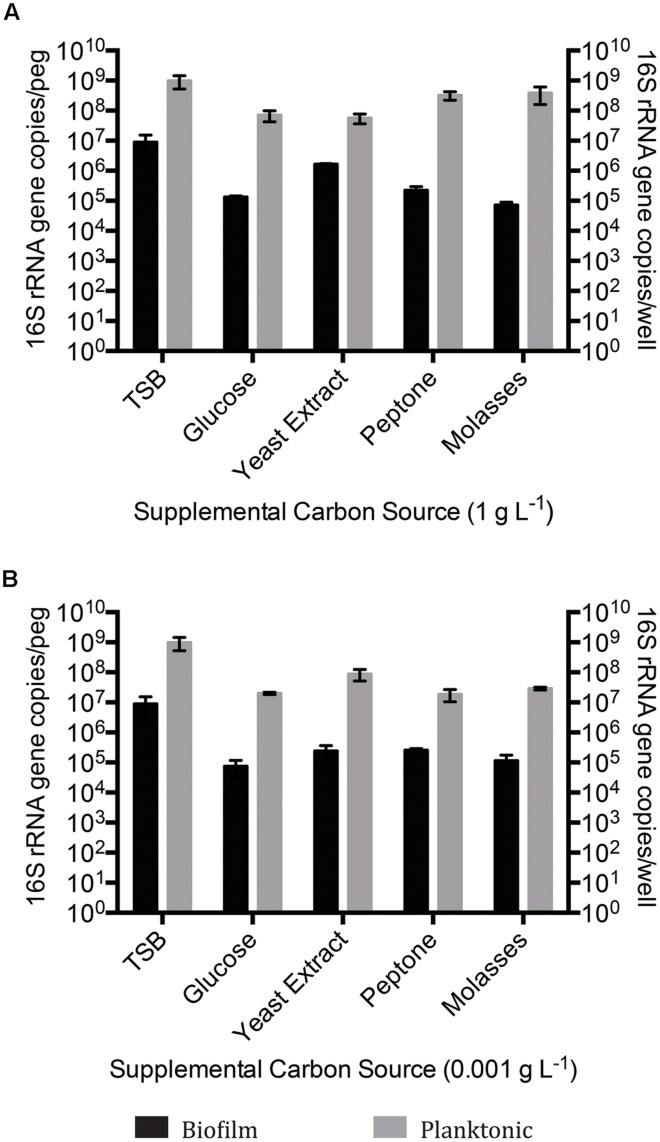
**Quantitative polymerase chain reaction (qPCR) 16S rRNA gene enumeration of mixed species biofilm and planktonic cultures grown for 14 days on BH minimal medium in the presence of 8χNA and either 1 g L^-1^ supplemental carbon **(A)**, or 0.001 g L^-1^ supplemental carbon **(B)**.** Enumeration of biofilms is reported as 16S gene copies per peg, enumeration of planktonic microbes reported as copies per well (*n* = 3). Error bars represent standard error of the mean.

Prior to further study, the existence of viable microbes within the OSPW sample was confirmed by inoculating the rich TSB medium directly with OSPW followed by subsequent aerobic incubation in the chambers of a microtiter plate or CBD for 14 days at 25°C, and 125 rpm. The resulting multispecies biofilms were discovered via confocal microscopy to be full and confluent (**Figure 1A**). Enumeration by qPCR revealed that TSB grown planktonic multispecies cultures were more numerous (10^9^ gene copies per well) than their biofilm counterparts (10^7^ gene copies per peg). Furthermore, TSB control cultures were more numerous than the cultures grown in the presence of 8χNA by up to two orders of magnitude (**Figure 2**).

When incubated with BH minimal medium in the presence of 8χNA, CLSM revealed thicker multispecies biofilms with better-developed microcolony structures when supplemental carbon sources were provided at 1 g L^-1^, as compared to those supplemented at 0.001 g L^-1^ (**Figure 1**). Quantification by qPCR corroborated these findings, demonstrating that 1 g L^-1^ supplemented multispecies biofilms are more populous than 0.001 g L^-1^ supplemented multispecies biofilms by roughly an order of magnitude (**Figure 2**). Multispecies OSPW planktonic cultures confirmed first via optical density measurement (data not shown), demonstrated the same trend; 1 g L^-1^ supplementation resulted in a more numerous planktonic culture as compared to 0.001 g L^-1^ supplementation by on average 1 order of magnitude (**Figure 2**). As in the viability control, multispecies planktonic cultures were more turbid than their biofilm counterparts by approximately 2 orders of magnitude (**Figure 2**).

**FIGURE 3:**
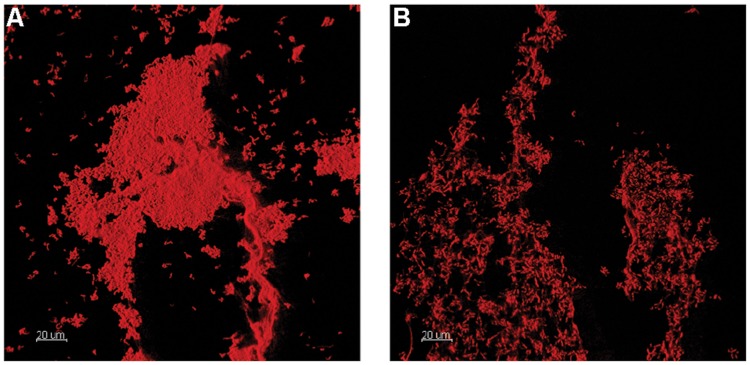
**Confocal laser scanning microscopy 3D renderings of OSPW single species *Rhodococcus* sp. (Y3; **A)**, and *Cyanobacteria* (G1; **B)** biofilms grown on the polystyrene pegs of the CBD for 14 days at 25°C, 125 rpm using BH minimal medium supplemented with either yeast extract or glucose (at 1 g L^-1^) respectively**.

**FIGURE 4:**
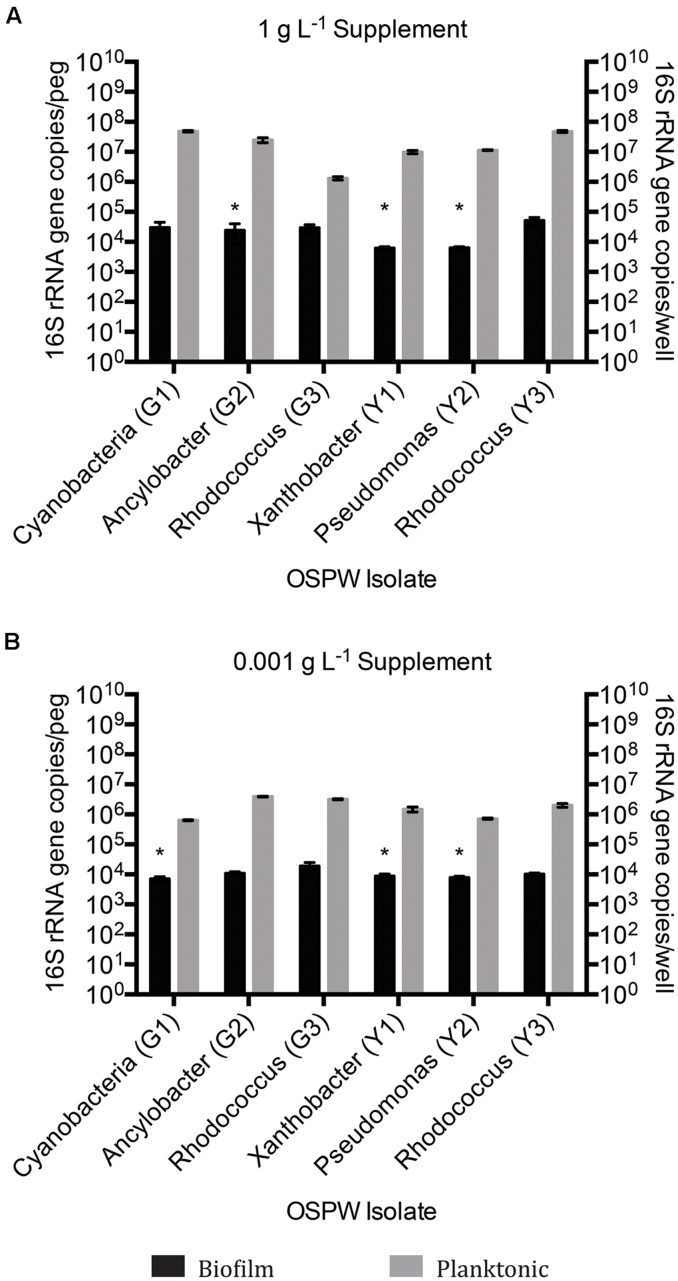
**qPCR 16S rRNA gene enumeration of single species biofilm and planktonic isolates grown for 14 days on BH minimal medium in the presence of 8χNA and either 1 g L^-1^ supplemental carbon **(A)**, or 0.001 g L^-1^ supplemental carbon **(B)**.** Enumeration of biofilms is reported as 16S gene copies per peg, enumeration of planktonic microbes reported as copies per well (*n* = 3). Error bars represent standard error of the mean. Asterisks denote samples whose qPCR copy number threshold was less than or equivalent to non-template controls.

Single species OSPW cultures were capable of growing in the presence of 8χNA as biofilms, evidenced by CLSM (**Figure 3**), and as planktonics, evidenced by optical density (data not shown), but were slower growing under these conditions than multispecies cultures. The 14 days incubation period was insufficient for biofilm growth to exceed 10^4^ copies per peg, regardless of the concentration of nutrient amendment (**Figure 4**). This proved problematic for select isolates, as the sensitivity of the qPCR assay is less robust at lower copy numbers. This is highlighted in **Figure 4** by the presence of asterisks above isolates whose growth was near the detection limits of the qPCR assay following an evaluation of the sample and non-template control qPCR copy threshold values as described elsewhere (Smith et al., 2008; Smith and Osborn, 2009). When grown planktonically, the six OSPW isolates achieved microbial populations on the order of approximately 10^8^ or 10^6^ gene copies per well depending on supplemental nutrient concentration (1 or 0.001 g L^-1^ respectively; **Figure 4**).

Regardless of whether cultures are mixed or single species, the 16S rRNA gene qPCR data demonstrated that planktonically grown cultures were up to several orders of magnitude more populous than corresponding biofilm cultures (**Figures 2** and **4**).

### Multispecies 8χNA Degradation Assay

**FIGURE 5:**
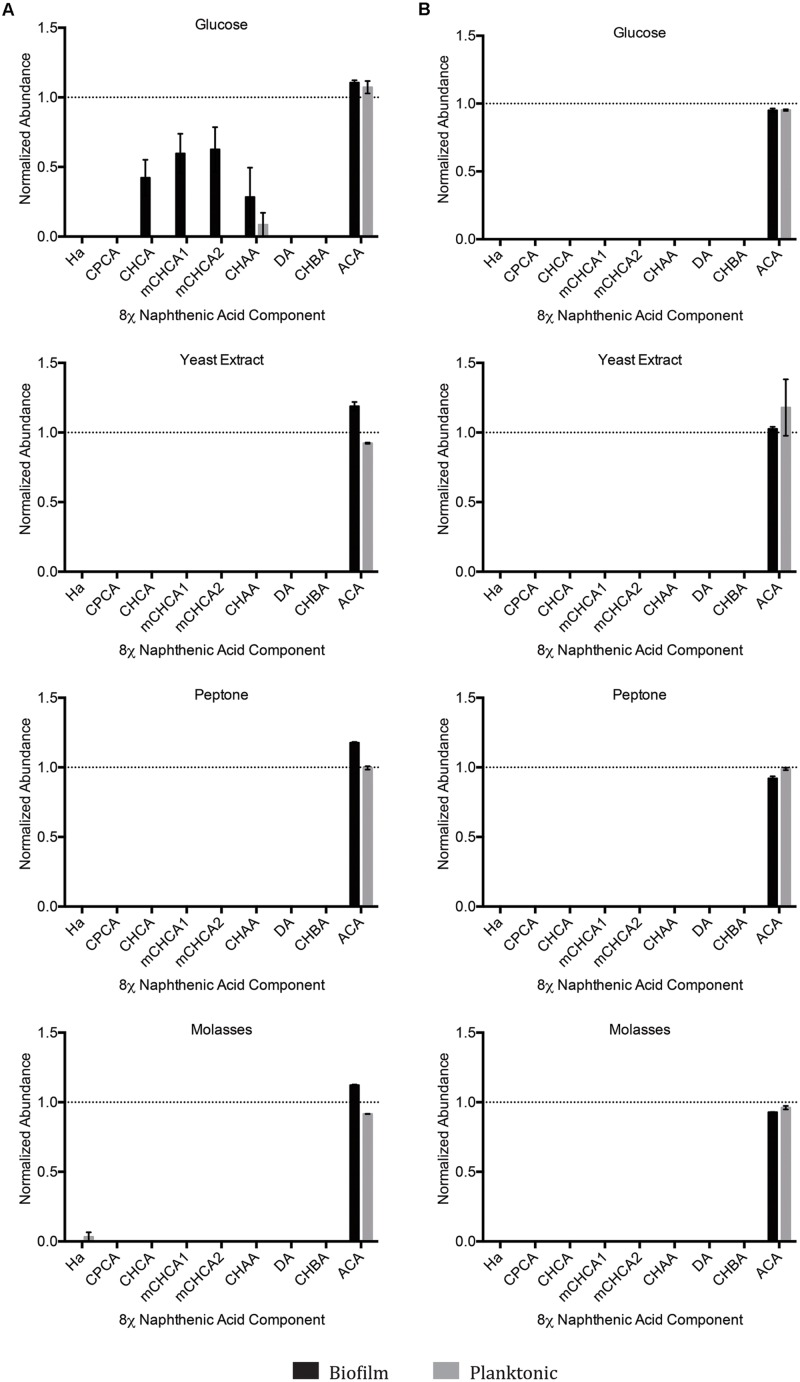
**Abundance of individual components of the 8χNA mixture following 14 days incubation with OSPW multispecies biofilm and planktonic cultures grown in BH minimal medium with either 1 g L^-1^ supplemental carbon **(A)**, or 0.001 g L^-1^ supplemental carbon **(B)**.** GC-FID was used to determine normalized abundance values relative to an internal standard (*n* = 2). Error bars represent standard error of the mean. A normalized abundance value of ‘1.0’ represents starting NA levels.

Upon organic extraction of the NAs remaining in the culture medium (both biofilm and planktonic), the presence of any remaining constituent NAs from the 8χNA mixture was evaluated by use of GC-FID instrumentation. The abundance (relative to the 4-PBA internal standard) of each NA was calculated, and normalized such that 1.0 abundance units represents individual NA starting levels. Note that 3-methyl-1-cyclohexane carboxylic acid (mCHCA) *cis* and *trans* stereoisomers separate in the GC column, and thus elute at different times, resulting in two separate peaks for mCHCA. Under all concentrations and permutations of supplemental carbon sources, both biofilm and planktonic OSPW multispecies cultures demonstrated complete degradation (below detectable limits) of all 8χNA constituent NAs within the 14 days incubation period, save for two noteworthy exceptions (**Figure 5**). First, not one OSPW multispecies culture exhibited any degradation of ACA. Secondly, when supplemented with 1 g L^-1^ glucose, multispecies biofilms did not degrade CHCA, mCHCA (both isomers) and CHAA below detectable limits; identical planktonic cultures also failed to degrade CHAA to below detectable limits (**Figure 5**).

### Single Species 8χNA Degradation Assays

**FIGURE 6:**
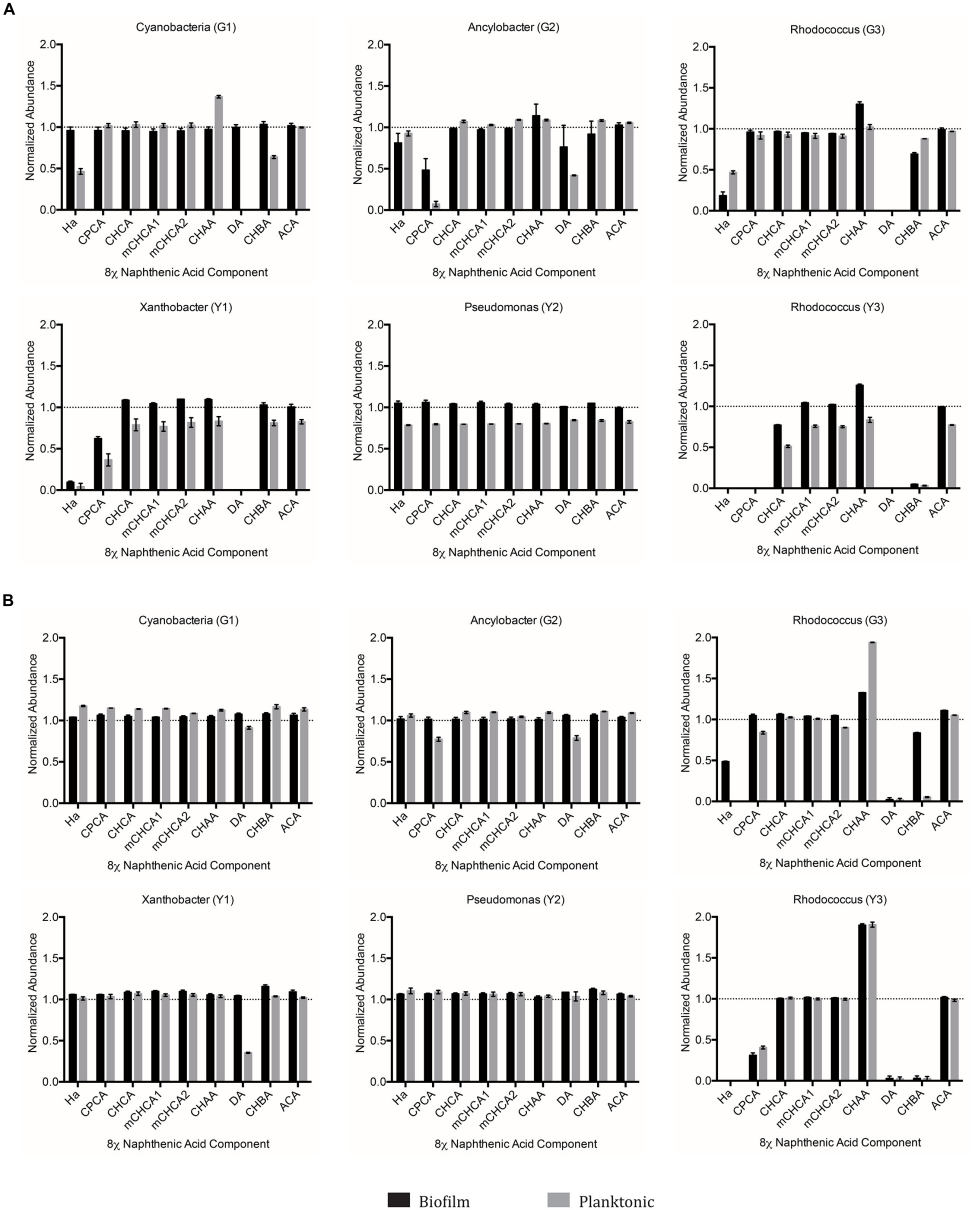
**Abundance of individual components of the 8χNA mixture following 14 days incubation with OSPW single species biofilm and planktonic isolates grown in BH minimal medium with either 1 g L^-1^ supplemental carbon **(A)**, or 0.001 g L^-1^ supplemental carbon **(B)**.** GC-FID was used to determine normalized abundance values relative to an internal standard (*n* = 2). Error bars represent standard error of the mean. A normalized abundance value of ‘1.0’ represents starting NA levels.

We anticipated that the single species isolates would struggle to degrade select components of the 8χNA tested here. As such, the single species isolates collectively may hold the potential to help elucidate structure-persistence relationships among the NAs chosen to comprise the 8χNA mixture. The data provided by the single species 8χNA degradation assay reveals that no single isolate was able to degrade as many NAs below detectable limits as multispecies cultures (**Figure 6**). Different isolates proved capable of degrading different NAs, and were influenced in various ways by high and low concentrations of supplemental carbon sources (either glucose or yeast extract). As such, a close examination of each OSPW isolate is warranted.

When grown as a biofilm under either concentration of glucose, the *Cyanobacteria* (G1) isolate failed to degrade any of the 8χNA components. In contrast, planktonic *Cyanobacteria* (G1) cultures demonstrated degradation of hexanoic acid (HA), decanoic acid (DA), and cyclohexane butyric acid (CHBA), but only when amended with 1 g L^-1^ glucose (**Figure 6**). *Ancylobacter* sp. (G2) biofilm and planktonic cultures exhibited degradation of cyclopentane carboxylic acid (CPCA) and DA when amended with 1 g L^-1^ glucose. Under 0.001 g L^-1^ amendments, only planktonic cultures were able to slightly degrade CPCA and DA (**Figure 6**). *Rhodococcus* sp. (G3) differed from the other glucose-OSPW isolates in that 0.001 g L^-1^ glucose amendment arguably resulted in better NA degradation, especially for CHBA. Moreover, the biofilm 8χNA degradation profile mimicked that of the planktonic state. Regardless of the concentration of glucose provided, this isolate displayed degradation of HA, DA, and CHBA. Conversely, this degradation assay demonstrated an increase in CHAA, particularly by 0.001 g L^-1^ glucose amended planktonic cultures (**Figure 6**).

When *Xanthobacter* sp. (Y1) was amended with 0.001 g L^-1^ yeast extract, only planktonic cultures showed degradation of just DA alone. Degradation of HA, DA, and CPCA proved possible by both biofilm and planktonic cultures if this isolate was incubated with yeast extract at 1 g L^-1^ (**Figure 6**). The *Pseudomonas* sp. (Y2) failed to conclusively indicate degradation of any 8χNA constituent NA as either a biofilm or planktonic culture, with either high or low concentrations of yeast extract (**Figure 6**). Lastly, the *Rhodococcus* sp. (Y3) demonstrated a similar 8χNA degradation profile as *Rhodococcus* sp. (G3), readily degrading HA, DA, CPCA, and CHBA, while producing a build-up of CHAA, as both a biofilm and planktonic culture under either concentration of yeast extract. It may also be cautiously interpreted that 1 g L^-1^ yeast extract amended cultures of this isolate were capable of degrading CHCA (**Figure 6**).

### Targeted ACA Degradation Assay

**FIGURE 7:**
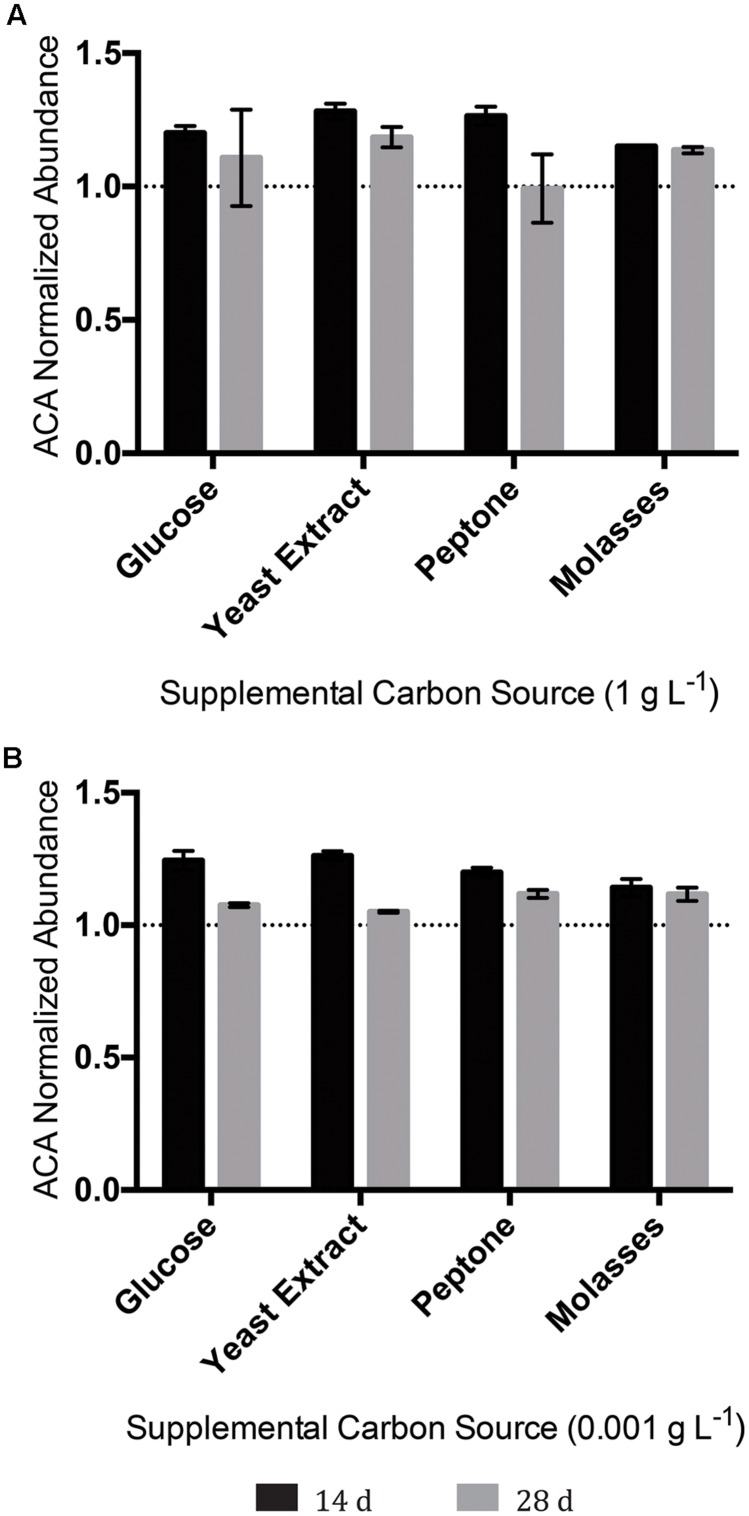
**Abundance of the tricyclic, diamondoid ACA following incubation with OSPW multispecies biofilms grown in BH minimal medium and 100 mg L^-1^ ACA with either 1 g L^-1^ supplemental carbon **(A)**, or 0.001 g L^-1^ supplemental carbon **(B)**.** GC-FID was used to determine normalized abundance values relative to an internal standard at 14, and 28 days time points (*n* = 2). Error bars represent standard error of the mean. A normalized abundance value of ‘1.0’ represents starting ACA levels.

Since no system tested had resulted in any discernable degradation of the tricyclic diamondoid NA ACA, we conducted an experiment aimed at achieving ACA degradation. To this end, OSPW multispecies biofilms were inoculated/incubated with 100 mg L^-1^ ACA as the sole NA provided, and evaluated (via GC-FID) for ACA degradation twice over a 28 days incubation period. Despite this comparatively extensive incubation period, no permutation of OSPW multispecies biofilm enriched with any concentration of supplemental carbon resulted in any discernable degradation of ACA (**Figure 7**).

For all mixed and single species experiments, sterile assays confirmed the absence of abiotic NA loss, confirming that observed losses are biotic in nature. Sterile NA levels post-incubation match those of day 0 controls (data not shown).

## Discussion

### Growth Response of OSPW Microbes to 8χNA

Since the indigenous OSPW microbes have been exposed to naturally occurring NAs, it was reasonable to presume that within the OSPW population existed microbes capable of surviving, and even utilizing NAs as a carbon or energy source. This holds true, as evidenced in **Figures 1–4** by the existence of microbial growth (as both mixed and single species biofilm and planktonic cultures) in the presence of 8χNA. Despite having been isolated from OSPW, and incubated under identical conditions, single species isolates do not grow as densely as biofilm and planktonic cultures as the multispecies cultures (**Figures 2** and **4**). One possible explanation for this may be the enhanced microniche exploitation of multispecies cultures compared to single species. Within a biofilm, chemical heterogeneity creates spatially segregated nutrient conditions or ‘microniches’ (Scheffer and van Nes, 2006), which are consequently populated by various microbial species with unique metabolic requirements and functions tailored to each microniche (Stewart and Franklin, 2008). Presumably, microniche exploitation by multiple different microbial species maximizes nutrient utilization and thus colonization of the environment (Stoodley et al., 2002). Spatially segregated microniches should not form in a planktonic culture if sufficient mixing is present. This does not, however, rule out temporal changes in the nutrients available to a planktonic culture. A single species planktonic batch culture in theory would have a limited metabolic potential and would be expected eventually to deplete its limiting resource and reach a stationary phase. Alternatively, multispecies cultures may be more populous than single species cultures as a result of beneficial, metabolic social interactions between different microbes (described in greater detail in the following subsection).

Another important parameter evaluated in this study was the form, and concentration of supplemental carbon sources provided. Here, we tested the degradation of the 8χNA mixture in the presence of either high or low concentrations of commercially available, supplemental carbon sources. We hypothesized that an additional carbon source may be necessary to maintain microbial diversity, which may help to promote favorable coordinated metabolic activities, and prevent community fixation as the result of over-enrichment (a situation in which the community becomes over-specialized and can no longer adapt to changing abiotic conditions; Stach and Burns, 2002). For example, it is plausible that growth on 8χNA alone may result in a fixated population no longer capable of adapting to, and degrading other OSPW NAs, should this community be used to treat OSPW. As seen in **Figures 2** and **4**, adding more easily amenable carbon sources results in a more populous culture overall. The presence of readily utilized nutrients may be important to not only maintain microbial population size, but may also serve a role in maintenance of xenobiotic degradative enzymes (Allard and Neilson, 1997). Conversely, too much amenable carbon may actually hinder the culture’s ability to degrade the xenobiotic in question, which may explain why 1 g L^-1^ glucose amended mixed species cultures exhibited less 8χNA degradation (**Figure 5A**; Leddy et al., 1995). One study found that a certain level of nutrient depletion was required to increase synthesis of catabolic enzymes, and thus maximize naphthalene degradation (Guerin and Boyd, 1995); hence the importance of testing 8χNA degradation at both high (1 g L^-1^) and low (0.001 g L^-1^) concentrations of supplemental carbon.

The generalized observation that planktonic cultures are more populous than corresponding biofilm cultures should be interpreted with caution, as this was most likely an artifact of the design of the 16S rRNA gene assay. In planktonic cultures, the entire microbial contents of each well were assayed for the presence of 16S rRNA genes, whereas in biofilm cultures only cells adhered to the CBD polystyrene peg were assayed; planktonic cells shed from the biofilm (which add to the total well microbial contents) were ignored.

### Microbial Sociality and Community Interactions Implicated to Confer Greater NA Degradation Capabilities

The results of our study suggest that harboring multiple microbial species within a consortium was advantageous to the degradation capacity of NAs, as evidenced by the fact that not one single species isolate could replicate the more substantial NA degradation trends of the mixed species community (**Figures 5** and **6**). The enhanced proficiency of mixed species OSPW cultures to degrade the 8χNA mixture correlates well with other studies examining organic pollutant degradation. Our prior NA work demonstrated that multispecies OSPW cultures could degrade both CHCA and CHAA, whereas only one of our OSPW isolates was capable of degrading CHCA, but not CHAA (Demeter et al., 2014). Del Rio et al. (2006), observed far greater degradation of a commercial NA mixture (Kodak) when they co-cultured their two pseudomonads. In a study of PAH degradation, it was discovered that a mixed culture of four bacterial isolates were better at degrading a defined PAH mixture than any of the four isolates individually (Trzesicka-Mlynarz and Ward, 1995). In this particular study we compared an OSPW representative community (mixed species cultures) to a total of six individual OSPW isolates. No attempts were made to evaluate co-cultures of any of the six isolates for a number of reasons. Successful co-culturing of isolates toward generating a specific mixed species biofilm is not a trivial matter. Careful consideration, a well-designed inoculation schedule, and the ability to conclusively detect each isolate is necessary to ensure that all isolates are present within the biofilm, and that no isolates are undesirably eliminated (Schwering et al., 2013). Moreover, metagenomic analysis has revealed that there are thousands of OTUs present within OSPW (An et al., 2013). Co-culturing of a mere six isolates therefore would not be representative of the OSPW community; direct growth of the OSPW community in our CBD system as a mixed species biofilm allowed us to overcome that hurdle.

Although not explicitly examined in our current study, alternative work suggests the causative phenomenon responsible for the enhanced NA degradation by mixed species cultures may be social, community level interactions. For example, Cowan et al. (2000) exemplify this well in their study of organic pollutant degradation, whereby *p-*cresol metabolizing *Pseudomonas putida*, and 2-chloroethanol metabolizing *Pseudomonas* sp. mixed species biofilms were able to degrade both of these compounds concurrently, but were unable to metabolize either compound as a single species biofilm. The presence of the other compound in the waste stream inhibited the degradation of the compound for which the bacterium was capable of metabolizing (Cowan et al., 2000). Such may be the case for our *Pseudomonas* sp. (Y2) OSPW isolate, which has previously demonstrated the capacity to degrade CHCA in the presence of CHAA (Demeter et al., 2014), but was unable to do so in the presence of the complete 8χNA mixture (**Figure 6**).

In a more sophisticated manner, heightened microbial diversity within close physical proximity may lead to beneficial or cooperative social interactions between members of the community (Mitri et al., 2011; Nadell et al., 2013). For example, one microbe may use the metabolic waste product of a species from a nearby microniche resulting in metabolic cross feeding. Such metabolic complementation is often necessary to metabolize recalcitrant xenobiotic compounds, as it prevents the buildup of potentially toxic intermediates, and promotes substrate mineralization (Field et al., 1995; Stach and Burns, 2002; Edwards and Kjellerup, 2013).

### Structure-Persistence Relationships of NAs

The mechanism(s) responsible for NA biodegradation have been elucidated in several studies, and reveal the preferred pathway to be via β-oxidation initiated at the carboxylic acid functional group (Taylor and Trudgill, 1978; Smith et al., 2008; Jones et al., 2011b). Studies have successfully linked NA structure to degradability, generating a NA structure-persistence relationship. Aside from even numbered carboxylic acid functional groups, studies have shown that alkyl side chain branching hinders NA degradation (Herman et al., 1993; Smith et al., 2008; Johnson et al., 2011; Huang et al., 2012), as well as an increase the degree of cyclicity (ring cleavage is energetically costly) and molecular weight (Scott et al., 2005; Han et al., 2008; Toor et al., 2013; Misiti et al., 2014). Other recalcitrant NAs include non-classical NAs, such as those containing elements of S or N (Headley et al., 2010).

Naphthenic acid structure-persistence relationships may explain many of the results observed for the OSPW single species 8χNA assays conducted in this study. For example, the *Pseudomonas* sp. (Y3) isolate was capable of degrading CHCA, which has an odd-length carboxylic acid functional group of one carbon, requiring ring cleavage upon the first successful round of β-oxidation (**Figure 6**). In contrast, CHBA (which was degraded by a total of three isolates) may undergo a first round of β-oxidation without requiring ring cleavage, resulting in a loss of the parent structure of CHBA. Not one OSPW isolate conclusively degraded CHAA (**Figure 6**), which was likely a result of the fact that it has an even-length carboxylic acid functional group of two carbons, and therefore a tertiary carbon on the ring, which blocks the removal of the acetate moiety via β-oxidation (Herman et al., 1993). More interesting, however, was the apparent buildup of CHAA in the culture media of the two Rhodococci (G3 and Y3; **Figure 6**). Theoretically, a loss of an acetate moiety from the carboxylic acid functional group of CHBA results in the production of the more recalcitrant CHAA as a metabolite; this has been observed in other studies evaluating CHBA degradation as well (Quesnel et al., 2011). The buildup of a degradation metabolite (such as CHAA in this case) provides further endorsement for the use of OSPW mixed species cultures, which were capable of degrading CHAA (along with all of the other constituent NAs used in this study, save for ACA; **Figure 5**). None of the OSPW isolates evaluated were capable of degrading either isomer of mCHCA under the conditions tested (**Figure 6**). This methylated form of CHCA was likely more recalcitrant because of it’s additional alkyl side chain (Smith et al., 2008).

Positively identified as a constituent of OSPW (Rowland et al., 2011a), tricyclic ACA displays the greatest degree of cyclicity of all the constituent NAs tested. More specifically, this NA is in fact a diamondoid NA with a complex structure of fused rings (Barker et al., 2000; Rowland et al., 2011b). Since ACA was not degraded by any single species isolate (**Figure 6**), or mixed community (with or without the presence of other NAs; **Figures 5** and **7**) we believe it is a fair assumption to label ACA as the most recalcitrant constituent of 8χNA. Additional pre-treatment methods such as ozonation may be required to render recalcitrant compounds such as ACA more labile prior to microbial degradation (Pérez-Estrada et al., 2011).

Although not included in the scope of this study, another important factor to consider is the achievement of overall toxicity goals of any potential NA/OSPW bioremediation effort. For example, the scaled bioreactors used in this study were unable to degrade ACA. At first glance this seems problematic, however, a recent NA toxicity study has found that tricyclic, diamondoid NAs (including ACA) are in fact some of the least toxic NA species (Jones et al., 2011a).

## Conclusion

This study sought to evaluate the hypothesis that mixed species biofilms are an effective strategy for harnessing microbes for use in remediation of OSPW, specifically the NA fraction, due to possible coordinate metabolic activity and heightened stressor tolerance in the biofilm state. Within the scope of this study, comparatively neither biofilm nor planktonic cultures proved significantly more effective than the other at degrading the NAs tested. Depending on reactor design, or presence of toxic co-contaminants, biofilms may yet prove more efficacious at degrading NAs, but not under the conditions tested here. Both biofilm and planktonic OSPW multispecies cultures were able to degrade seven of the eight NAs tested below detectable limits, whereas single species isolates (biofilm or planktonic) were only capable of degrading a small subset of NAs. This indicates the importance of a socially connected community of microbes. Multispecies cultures (such as those grown in our CBD system) may therefore be considered a choice method in which to harness OSPW microbes for *ex situ* NA bioremediation; the ability to harness a high proportion of the whole community is key. Lastly, the persistence of a compound such as ACA (or by extension persistent fractions in oil sands tailings acid extracted organics) demonstrates the need to consider a battery or series of various treatments to effectively remove such compounds.

## Conflict of Interest Statement

The authors declare that the research was conducted in the absence of any commercial or financial relationships that could be construed as a potential conflict of interest.
